# Cephalosporin nitric oxide-donor prodrug DEA-C3D disperses biofilms formed by clinical cystic fibrosis isolates of *Pseudomonas aeruginosa*

**DOI:** 10.1093/jac/dkz378

**Published:** 2019-09-17

**Authors:** Odel Soren, Ardeshir Rineh, Diogo G Silva, Yuming Cai, Robert P Howlin, Raymond N Allan, Martin Feelisch, Jane C Davies, Gary J Connett, Saul N Faust, Michael J Kelso, Jeremy S Webb

**Affiliations:** 1 National Biofilms Innovation Centre, University of Southampton, Southampton SO17 1BJ, UK; 2 Biological Sciences and Institute for Life Sciences, University of Southampton, Southampton SO17 1BJ, UK; 3 Molecular Horizons and School of Chemistry & Molecular Bioscience, University of Wollongong, NSW, 2522, Australia; 4 Illawarra Health & Medical Research Institute, Wollongong, NSW, 2522, Australia; 5 Faculty of Medicine and Institute for Life Sciences, University of Southampton and University Hospital Southampton NHS Foundation Trust, Southampton SO16 6YD, UK; 6 NIHR Southampton Clinical Research Facility and NIHR Southampton Biomedical Research Centre, University Hospital Southampton NHS Foundation, Southampton SO16 6YD, UK; 7 Cystic Fibrosis Trust Strategic Research Centre and National Heart and Lung Institute, Imperial College London, London SW3 6LY, UK

## Abstract

**Objectives:**

The cephalosporin nitric oxide (NO)-donor prodrug DEA-C3D (‘DiEthylAmin-Cephalosporin-3′-Diazeniumdiolate’) has been shown to initiate the dispersal of biofilms formed by the *Pseudomonas aeruginosa* laboratory strain PAO1. In this study, we investigated whether DEA-C3D disperses biofilms formed by clinical cystic fibrosis (CF) isolates of *P. aeruginosa* and its effect in combination with two antipseudomonal antibiotics, tobramycin and colistin, *in vitro.*

**Methods:**

β-Lactamase-triggered release of NO from DEA-C3D was confirmed using a gas-phase chemiluminescence detector. MICs for *P. aeruginosa* clinical isolates were determined using the broth microdilution method. A crystal violet staining technique and confocal laser scanning microscopy were used to evaluate the effects of DEA-C3D on *P. aeruginosa* biofilms alone and in combination with tobramycin and colistin.

**Results:**

DEA-C3D was confirmed to selectively release NO in response to contact with bacterial β-lactamase. Despite lacking direct, cephalosporin/β-lactam-based antibacterial activity, DEA-C3D was able to disperse biofilms formed by three *P. aeruginosa* clinical isolates. Confocal microscopy revealed that DEA-C3D in combination with tobramycin produces similar reductions in biofilm to DEA-C3D alone, whereas the combination with colistin causes near complete eradication of *P. aeruginosa* biofilms *in vitro*.

**Conclusions:**

DEA-C3D is effective in dispersing biofilms formed by multiple clinical isolates of *P. aeruginosa* and could hold promise as a new adjunctive therapy to patients with CF.

## Introduction

*Pseudomonas aeruginosa*, a ubiquitous Gram-negative opportunistic pathogen, is a major cause of morbidity and mortality in cystic fibrosis (CF). Although initial *P. aeruginosa* lung infection can often be eradicated, reinfection is common and the prevalence of chronic infection increases with age.^1^ Chronic lung infection is associated with the formation of biofilms, which, once established, are almost impossible to eradicate despite aggressive antibiotic treatment.^2^

Biofilms in the CF lung typically develop as aggregated communities of bacteria in a self-produced exopolysaccharide matrix that contains both bacterial and mammalian cell debris.^3^ Biofilms are highly tolerant towards the host immune system and are up to 1000 times more tolerant to antibiotic treatment than when in planktonic phases of growth. This increased tolerance to antimicrobials is multifactorial and arises from inactivation of antibiotics in the biofilm matrix by bacterial secreted products or matrix components, the formation of unfavourable pH and oxygen gradients within the biofilm that reduce antibiotic efficacy, and altered bacterial cell metabolism.^4–6^ Persistent *P. aeruginosa* infection results in the gradual destruction of lung tissue, progressive respiratory failure and premature death in the majority of CF patients.^7^

A putative anti-biofilm treatment strategy involves reducing the tolerance of *P. aeruginosa* cells to antibiotics by initiating their dispersal from the biofilm. Previous research has shown that low-dose nitric oxide (NO; ∼450 nM) causes the dispersal of *P. aeruginosa* biofilms, rendering cells more susceptible to antimicrobials.^8^ NO-induced dispersal is caused by a reduction in the intracellular level of the bacterial messenger molecule bis-(3′-5′)-cyclic dimeric guanosine monophosphate (c-di-GMP) through modulation of phosphodiesterase and diguanylate cyclase activities.^9^ A recent proof-of-concept clinical trial conducted by our group demonstrated that low-dose inhaled NO (10 ppm) as an adjunctive to antibiotic therapy reduces *P. aeruginosa* burden in the lungs of CF patients without adverse effects,^10^ although pseudomonal biofilms were re-established following cessation of treatment. NO gas delivery to individuals with CF is expensive and impractical for routine care. A drug formulation capable of delivering controlled low-dose NO to the biofilm is potentially a more practical way of achieving long-term benefits.

**Figure 1.**
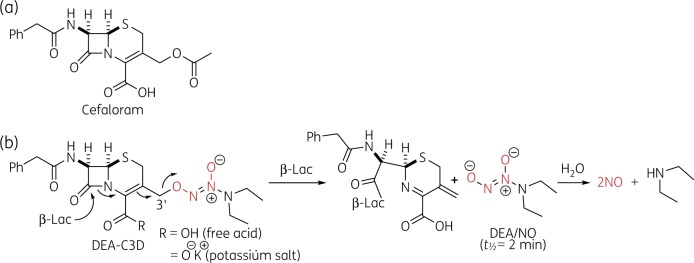
Chemical structures. (a) Structure of the first-generation cephalosporin antibiotic cefaloram. (b) Structure of DEA-C3D (free acid and potassium salt forms) and NO release mechanism following reaction with β-lactamases (β-Lac). This figure appears in colour in the online version of *JAC* and in black and white in the print version of *JAC*.

Cephalosporin-3′-diazeniumdiolates (C3Ds) are a recent class of NO-donor prodrugs designed to locally release NO following reaction of their β-lactam moiety with bacterial β-lactamases, thus triggering biofilm dispersion. Targeted release of NO at chronic infection sites by C3Ds could maximize NO delivery at the biofilm whilst minimizing host exposure to the reactive and potentially toxic NO. The prototypical example DEA-C3D (‘DiEthylAmin-Cephalosporin-3'-Diazeniumdiolate’) (Figure 1) contained the phenacetyl side chain of the first-generation cephalosporin cefaloram and the diazeniumdiolate NO donor DEA/NO (*t*_½_=2 min).^11^^,^^12^ The proposed mechanism of β-lactamase-triggered NO release and biofilm dispersion by DEA-C3D is shown in Figure 1. A related analogue, PYRRO-C3D, consisting of cefaloram linked to the faster acting diazeniumdiolate PYRRO/NO (*t*_½_=2 s), was recently shown to have activity against *Haemophilus influenzae* and *Streptococcus pneumoniae* biofilms.^13^^,^^14^

In our original reports on C3Ds, DEA-C3D was shown to disperse biofilms of *P. aeruginosa* PAO1, a common laboratory biofilm model.^11^^,^^12^ In this study, we investigated the ability of DEA-C3D to disperse biofilms formed by clinical CF isolates of *P. aeruginosa* and the anti-biofilm effects of its use in combination with the standard-of-care antipseudomonal antibiotics tobramycin and colistin.

## Materials and methods

### Bacterial strains and growth conditions

Bacterial strains used were *P. aeruginosa* PAO1 (University of Washington, USA) and three clinical isolates of *P. aeruginosa*, PA21, PA30 and PA68, isolated from the sputum of CF patients based at University Hospital Southampton NHS Foundation Trust (NHS Research Ethics Committee 08/H0502/126; patients aged 18, 19 and 51 years, respectively). PA21 and PA68 were from patients with chronic *P. aeruginosa* infection whilst PA30 was from a patient with intermittent *P. aeruginosa* infection; all three clinical isolates were from patients during a period of CF exacerbation. Overnight cultures were grown in LB broth at 37°C with agitation at 180 rpm. Biofilm cultures were cultivated at 37°C in M9 minimal medium (pH 7; Formedium) containing 48 mM Na_2_HPO_4_, 22 mM KH_2_PO_4_, 9 mM NaCl and 19 mM NH_4_Cl, and supplemented with 2 mM MgSO_4_, 100 μM CaCl_2_ and 20 mM glucose (all Sigma–Aldrich), with agitation at 50 rpm.

### DEA-C3D and antibiotics

DEA-C3D potassium salt and DEA-C3D free carboxylic acid were synthesized at the University of Wollongong, Australia, and stored at −80°C with desiccant. Details of synthesis for both DEA-C3D forms are described elsewhere.^11^^,^^12^ Stock solutions of DEA-C3D at 25.6 mM were prepared fresh in DMAO (Sigma–Aldrich) and diluted in medium immediately prior to use. Stock solutions of tobramycin and colistin sulfate (Sigma–Aldrich) were prepared in water at 20 mg/L and refrigerated or kept frozen until use.

### Evaluation of NO release

The release of NO from DEA-C3D was assessed using a highly sensitive chemiluminescence detector (CLD 88Y, Eco Physics), as previously described.^15^ Briefly, the set-up consists of a glass reaction vessel that is purged with a constant stream of carrier gas (300 mL/min) through a glass frit and connected to a scrubbing bottle containing 1 M NaOH to trap acid fumes and higher nitrogen oxides. NO generated in the reaction solution is continuously purged from the solution and transferred to the reaction chamber of the CLD where it reacts with ozone (O_3_). This reaction (see equations below) generates nitrogen dioxide in an excited state (NO_2_*), which emits light upon returning to the ground state.

(1) $$\text{NO}\;+{\;O}_{3}\to {{\;\text{NO}}_{2}}^{*}\;+{\;O}_{2}$$

(2) $${{\text{NO}}_{2}}^{*}\;\to {\;\text{NO}}_{2}+\;h\nu \;\left(\text{light}\right)$$

The light is then quantified by a photomultiplier tube and used to calculate the NO concentration. PBS was added to the reaction vessel (maintained at 37°C) while being continuously purged with air, and the baseline signal was recorded (<5 ppb NO). Medical-grade air (BOC) was used as a purge/carrier gas as all of the biological assays were carried out under aerobic conditions. DEA-C3D stocks in DMSO were prepared fresh, diluted with PBS and added to the reaction vessel to a final concentration of 10 μM, and *Bacillus cereus* penicillinase (Sigma–Aldrich; 20 U) was then added. NO readings were recorded using a PowerChrom data acquisition system (eDAQ), with each run being carried out in duplicate. For quantification of NO release over time, the area under the curve was integrated and concentrations extracted from an NO calibration curve, where known concentrations of NO were generated by nitrite reduction using the tri-iodide reagent, as previously described.^15^

### Planktonic susceptibility to DEA-C3D potassium salt, DEA-C3D carboxylic acid and cefaloram

The MICs of DEA-C3D potassium salt, DEA-C3D carboxylic acid and cefaloram for the planktonic cultures of four strains of *P. aeruginosa* were determined using the broth microdilution method. Briefly, a stock solution of the agent was added to LB broth and 2-fold serial dilutions created in a 96-well microtitre plate. After addition of a log phase inoculum in LB broth between 1 × 10^5^ and 5 × 10^5^ cfu/mL, plates were incubated at 37°C for 24 h. The MIC was identified as the lowest concentration required to inhibit bacterial growth, as determined by visual analysis.

### Microtitre plate assays to investigate effects of DEA-C3D potassium salt on clinical CF isolate biofilms of P. aeruginosa

The effects of DEA-C3D potassium salt on biofilms formed by clinical isolates of *P. aeruginosa* and PAO1 were analysed using the reported microtitre plate-based assay with some modifications.^16^^,^^17^ Overnight cultures were diluted 1 in 100 into freshly prepared M9 minimal medium and added into the non-perimeter wells of a flat-bottomed tissue culture-treated 96-well microtitre plate (Thermo Scientific). Perimeter wells were not utilized for biofilm growth as they represent the wells with the highest variability in biofilm growth rate. Uninoculated M9 minimal medium was added to the perimeter wells and these served as negative controls. Plates were then incubated with shaking at 50 rpm at 37°C for 24 h. Following incubation, the planktonic suspensions were gently aspirated using a pipette and the biofilms washed once with M9 minimal medium. Biofilms were then treated with DEA-C3D, as follows: a stock concentration of DEA-C3D in DMSO (25.6 mM) was diluted 1 in 100 in M9 minimal medium, to produce a final concentration of 256 μM DEA-C3D with 1% DMSO. This was then serially diluted in a new 96-well microtitre plate with M9 minimal medium containing 1% DMSO in a 2-fold manner (to maintain a consistent DMSO concentration across all DEA-C3D dilutions). Serial dilutions of DEA-C3D were then added to the washed biofilm cultures. M9 minimal medium containing only 1% DMSO was added to the wells of the untreated control biofilms. Following a 20 h treatment period, the planktonic suspensions were transferred to a new 96-well microtitre plate and the absorbance values were recorded at 584 nm with a FLUOstar Omega plate reader (BMG Labtech) to assess viability of the planktonic cultures. Biofilms were then washed twice with PBS, stained with 0.1% crystal violet for 15 min and then washed once more to remove excess stain. To extract the crystal violet back into solution, 30% acetic acid was added to the wells and left for 20 min at room temperature. The absorbance of the resolubilized crystal violet stain was then measured at 584 nm with the FLUOstar Omega plate reader (BMG Labtech).

### Microscopy of biofilms treated with DEA-C3D potassium salt and antibiotics

Confocal laser scanning microscopy (CLSM) was used to analyse the effects of DEA-C3D and antibiotics on *P. aeruginosa* strain PA68 biofilms. Overnight cultures were diluted 1 in 100 in M9 minimal medium and inoculated into 35 mm glass bottom microwell plates (MatTek Corporation). Plates were incubated at 37°C with gentle shaking at 50 rpm for 24 h and then treated with DEA-C3D and/or antibiotics for 20 h. Antibiotic concentrations were chosen based on preliminary experiments to establish concentrations that were sublethal to the biofilm. Following treatment, biofilms were washed twice with Hanks balanced salt solution to remove non-attached cells, and biofilm viability was assessed using the LIVE/DEAD BacLight Bacterial Viability Kit (Life Technologies) containing dyes SYTO9 and propidium iodide (PI), as per the manufacturer’s instructions. Biofilms were then examined with an inverted Leica SP8 confocal laser scanning microscope using the ×63 oil immersion lens with scanning at 1 μm intervals. Argon and DPSS laser lines at 488 nm and 561 nm were used for excitation and the FITC filter set cube was used to acquire images. Images were obtained and analysed using the LAS AF software (Leica Microsystems GmbH). Assays were performed using two technical replicates of two biological replicates (*n=*4); five images were taken from each replicate biofilm, in a pre-determined pattern to avoid selection bias. COMSTAT 2.0 software was used for quantitative analysis of images.^18^

### Statistical analysis

GraphPad Prism 7.0 was used to conduct statistical analysis. Specific tests are detailed in the figure captions. A *P* value of <0.05 was deemed significant. For all analyses: ns=non-significant, *=*P < *0.05, **=*P < *0.01, ***=*P < *0.001 and ****=*P < *0.0001.

## Results 

### DEA-C3D selectively releases NO in response to penicillinase

**Figure 2.**
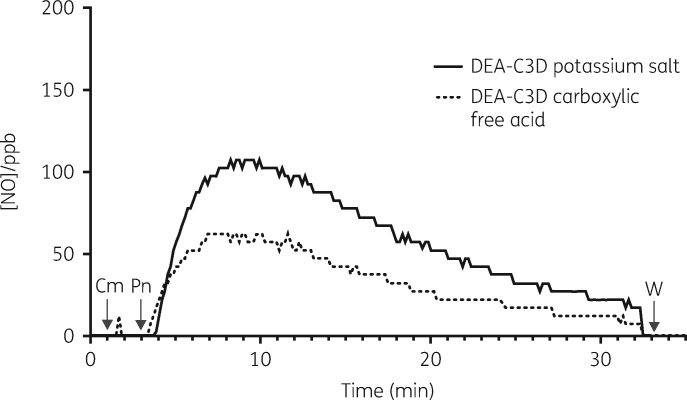
Representative traces showing NO release from DEA-C3D (potassium salt and carboxylic acid) upon exposure to penicillinase. The release of NO was followed in real time by gas-phase chemiluminescence, with concentrations expressed in parts per billion (ppb). Arrows denote the time the compounds (Cm) and 20 U of *B. cereus* penicillinase (Pn) were added to the reaction chamber. Readings were discontinued 30 min after addition of penicillinase, at which time the reaction chamber was washed (W) and prepared for the next analysis. Experiments were carried out in duplicate and showed similar results.

A highly sensitive chemiluminescence detection (CLD) method was used to confirm β-lactamase-triggered release of NO from DEA-C3D (Figure 2). The DEA-C3D potassium salt and carboxylic free acid forms both exhibited a similar NO release profile, showing minimal or no spontaneous release of NO in buffer alone and profound NO release within 1 min following addition of penicillinase. Peak NO release rates occurred at ∼5 min, after which both compounds displayed a gradual decline in NO levels over time. Total amounts of NO released from each compound (in moles) were calculated by comparing with peak areas of NO generated from sodium nitrite standards injected into a reducing reaction solution. Per 1 mL of a 10 μM solution (10 nmol), DEA-C3D carboxylic acid produced an average of 3.92 nmol of NO, whilst DEA-C3D potassium salt produced almost twice as much (an average of 6.82 nmol). This corresponded to an approximate NO yield of 40% and 70%, respectively, within 30 min.

### DEA-C3D lacks direct antibacterial activity against P. aeruginosa

MIC assays were carried out to measure the direct antibacterial activity of DEA-C3D potassium salt, DEA-C3D carboxylic free acid and cefaloram. The compounds were tested against PAO1 and three clinical CF isolates (PA21, PA30 and PA68). For all four *P. aeruginosa* strains there was no attainable MIC value for either DEA-C3D form, with bacterial growth observed at all concentrations up to 128 μM.

### Biofilms formed by multiple clinical CF isolates of P. aeruginosa are dispersed by DEA-C3D

**Figure 3.**
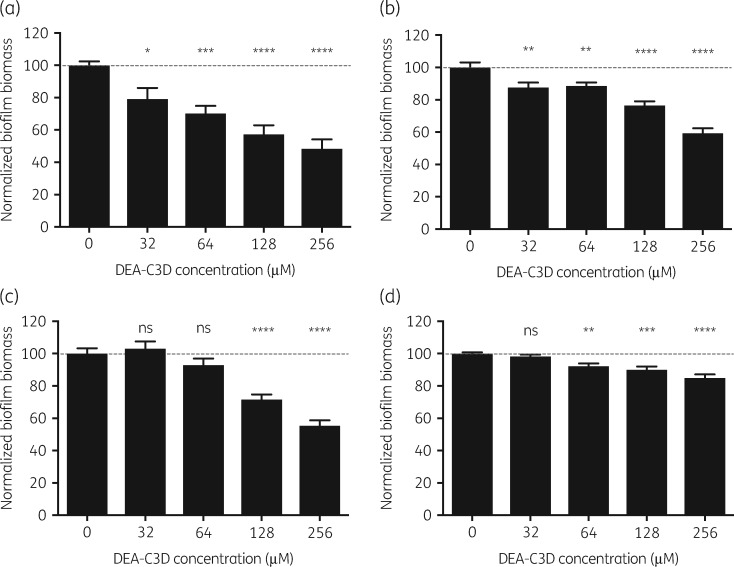
Dispersal of *P. aeruginosa* biofilms by DEA-C3D potassium salt. (a) PAO1 and three clinical CF isolates of *P. aeruginosa*: (b) PA21; (c) PA30; and (d) PA68. Biofilms were treated with DEA-C3D potassium salt before staining with crystal violet and extraction into acetic acid for spectrophotometric quantification. Results represent the mean±SEM from two independent experiments, each with six technical replicates (*n=*12). Ordinary one-way ANOVA with Dunnett’s multiple comparisons test was used for statistical analyses and each treatment group was compared with the untreated control. Values were normalized to 100 according to the untreated control for each isolate.

Crystal violet assays were used to evaluate the effects of DEA-C3D potassium salt on biofilms formed by the laboratory strain PAO1 and three clinical CF isolates of *P. aeruginosa* (Figure 3). For PAO1, DEA-C3D produced a dose-dependent reduction in biofilm biomass. At the highest concentration (256 μM), the mean biofilm biomass for PAO1 was reduced by 52% (*P*<0.0001) compared with untreated controls. DEA-C3D also dispersed biofilms formed by all three CF isolates in a dose-dependent manner, although the extent of dispersal was strain dependent. At the highest concentration (256 μM), DEA-C3D reduced the biofilm biomass of PA21, PA30 and PA68 by 41% (*P*<0.0001), 45% (*P*<0.0001) and 15% (*P*<0.0001), respectively, compared with the untreated controls for each isolate. Increased turbidity of the respective planktonic suspensions confirmed that DEA-C3D was causing biofilm dispersal, as opposed to exerting a direct antibacterial effect (see Figure S1, available as Supplementary data at *JAC* Online).

### DEA-C3D reduces biofilm biomass, thickness and surface area coverage of biofilms formed by P. aeruginosa clinical CF isolate PA68 alone and in combination with tobramycin

**Figure 4.**
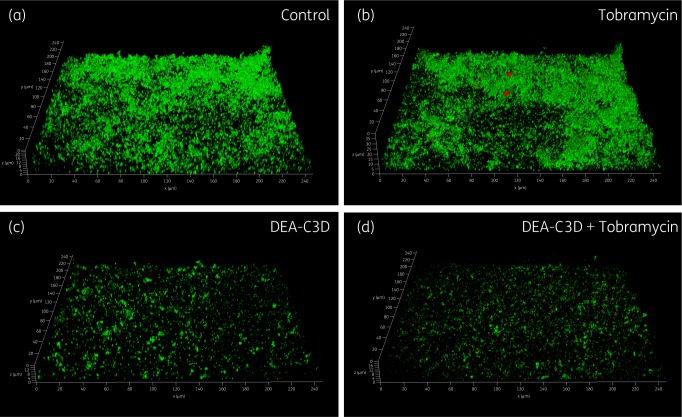
Appearance of *P. aeruginosa* biofilms following tobramycin and DEA-C3D treatments, alone and in combination. Biofilms formed by *P. aeruginosa* clinical CF isolate PA68 were treated with 4 mg/L tobramycin (b), 256 μM DEA-C3D potassium salt (c) or DEA-C3D/tobramycin combination (d), and compared with untreated control biofilms (a). Biofilms were stained with SYTO9 (green=viable cells) and propidium iodide (red=dead/damaged cells) before CLSM. Representative 3D images of each treatment are shown; *x*- and *y*-axes=246 μm by 246 μm. Experiments were carried out in duplicate and showed similar results.

CLSM was used to further probe the effects of DEA-C3D potassium salt on *P. aeruginosa* biofilms and to investigate the effects of co-treatment with tobramycin. Clinical isolate PA68 was chosen for these assays as it demonstrated robust and reproducible biofilm growth *in vitro*. Treatment with DEA-C3D (256 μM) visibly reduced the amount of biofilm formed by PA68 compared with untreated controls (Figure 4). Treatment with tobramycin alone (4 mg/L) showed no apparent effect, whereas co-treatment with DEA-C3D and tobramycin reduced the biofilm to a similar extent to DEA-C3D alone.

**Figure 5.**
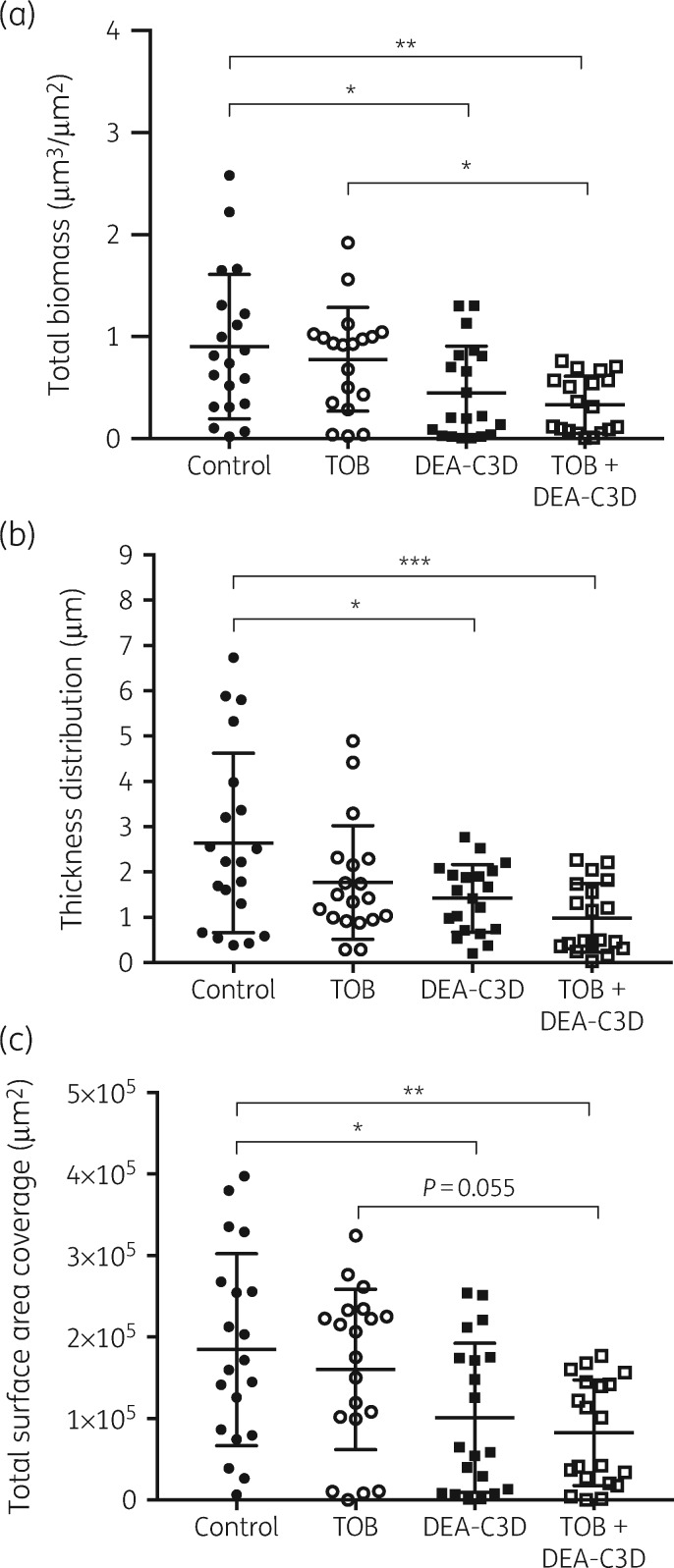
DEA-C3D potassium salt causes a reduction in *P. aeruginosa* clinical CF isolate PA68 biofilms alone and in combination with tobramycin. (a) Total biofilm biomass, representing live and dead biomass combined. (b) Biofilm thickness distribution. (c) Total biofilm surface area coverage. Values for all parameters were obtained from COMSTAT analysis of CLSM images. Scatter plots show all data points from *n*=4, with the mean and SD. One-way ANOVA with Tukey’s multiple comparisons tests were used for statistical analyses. TOB, tobramycin.

Quantifiable biofilm parameters supported the visual findings. DEA-C3D-treated biofilms showed a statistically significant reduction in total biofilm biomass, biofilm thickness distribution and total biofilm surface area coverage compared with untreated controls (Figure 5). Biofilms treated with tobramycin showed no effect in all three markers, whereas the combination of DEA-C3D and tobramycin produced statistically significant reductions compared with the untreated controls.

The mean total biofilm biomass was reduced by 50.2% by DEA-C3D (*P=*0.0069) and by 65.1% by the DEA-C3D/tobramycin (*P=*0.0006) combination compared with the untreated controls, though there was no significant difference between these two treatment groups (*P=*0.4122). Compared with tobramycin-treated biofilms, the combination treatment produced a 57.3% reduction in mean biofilm biomass (*P=*0.0453), though mean thickness distribution and surface area coverage did not demonstrate a significant change (*P=*0.2471 and *P=*0.0550, respectively). There was no statistically significant difference for any of the quantifiable parameters between biofilms treated with DEA-C3D alone or the DEA-C3D/tobramycin combination.

### DEA-C3D in combination with colistin results in almost complete eradication of biofilms formed by P. aeruginosa clinical CF isolate PA68

**Figure 6.**
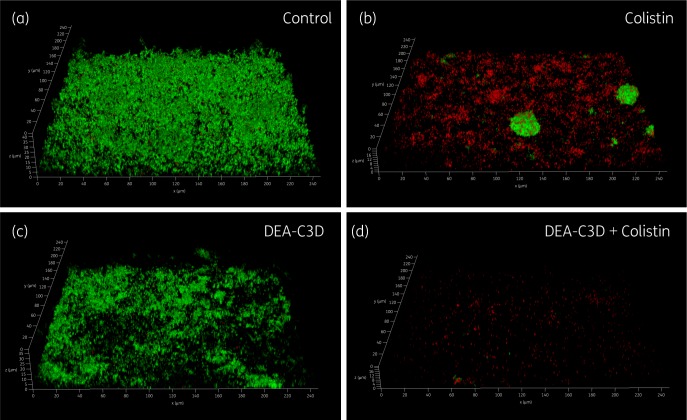
DEA-C3D enhances the activity of colistin against *P. aeruginosa* biofilms. Biofilms formed by clinical CF isolate PA68 were treated with 16 mg/L colistin (b), 256 μM DEA-C3D potassium salt (c) or DEA-C3D/colistin combination (d), and stained with SYTO9 (green=viable cells) and propidium iodide (red=dead/damaged cells) before imaging by CLSM. Untreated control biofilms are shown in (a). Representative 3D images for each treatment are shown; *x*- and *y*-axes=246 μm by 246 μm. Experiments were carried out in duplicate and showed similar results.

CLSM was used to investigate the anti-biofilm effects of DEA-C3D potassium salt in combination with colistin. Treatment of biofilms with colistin alone produced a visible effect, reducing both the amount of biofilm present and the number of viable cells. However, large ‘microcolonies’ consisting mostly of live *P. aeruginosa* cells still remained (Figure 6). DEA-C3D alone appeared to cause a visible reduction, but the effect of the combination treatment was much more striking, with virtually no viable cells remaining and almost all of the biofilm removed from the substratum. These observations were seen consistently across replicates; see the Supplementary data for the maximum projection images (cumulative z stacks) of all replicate images for untreated control biofilms (Figure S2), DEA-C3D-treated biofilms (Figure S3), colistin-treated biofilms (Figure S4) and DEA-C3D/colistin-treated biofilms (Figure S5).

**Figure 7.**
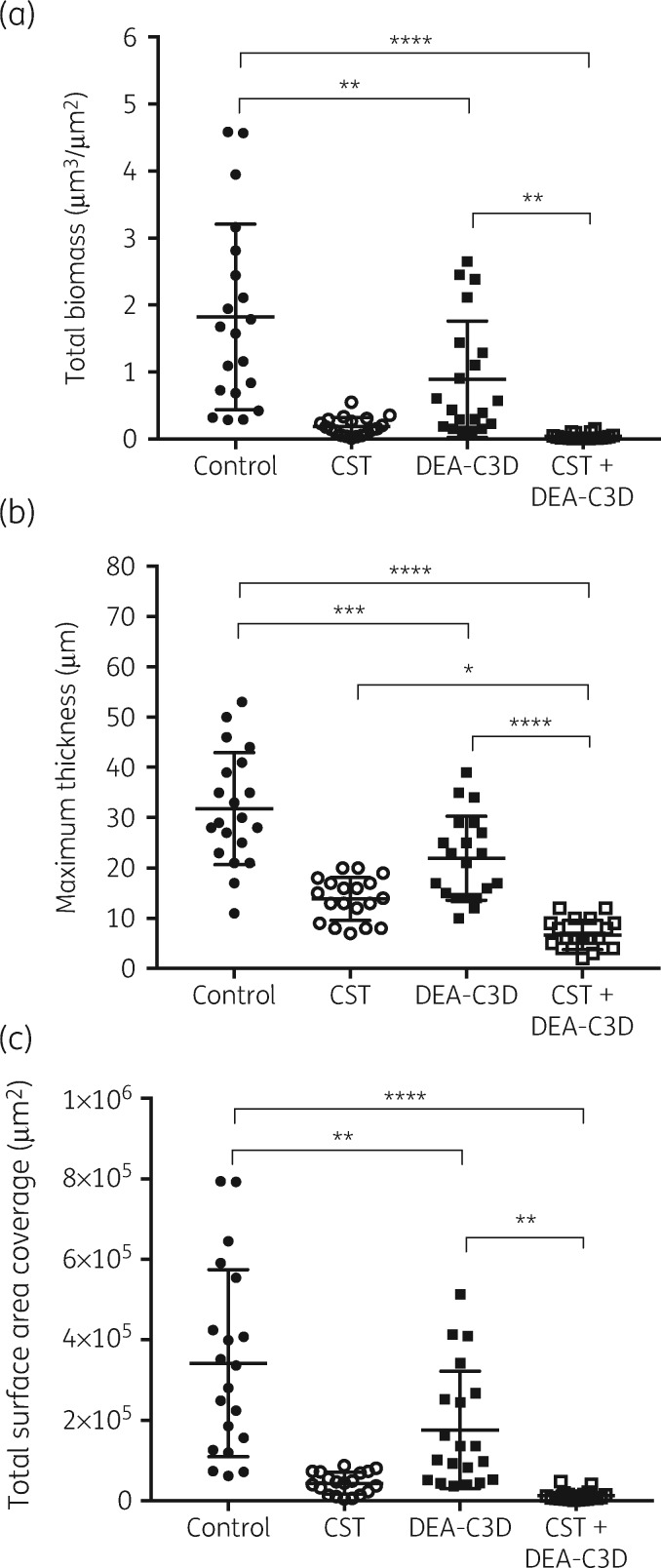
DEA-C3D potassium salt causes a reduction in *P. aeruginosa* clinical CF isolate PA68 biofilms alone and in combination with colistin. (a) Total biofilm biomass, representing live and dead biomass. (b) Maximum biofilm thickness. (c) Total biofilm surface area coverage. Values for all parameters were obtained from COMSTAT analysis of CLSM images. Scatter plots show all data points from *n*=4, with the mean and SD. One-way ANOVA with Tukey’s multiple comparisons tests were used for statistical analyses. CST, colistin.

The quantifiable biofilm parameters supported the visual observations. DEA-C3D-treated biofilms caused statistically significant reductions in total biofilm biomass, maximum biofilm thickness and total biofilm surface area coverage compared with untreated controls (Figure 7). The mean of all three parameters was significantly reduced in biofilms treated with the DEA-C3D/colistin combination compared with both untreated control biofilms and biofilms treated with DEA-C3D only. Compared with the untreated controls, the mean total biofilm biomass was reduced by 50.9% with DEA-C3D treatment (*P=*0.0036), by 89.8% with colistin treatment (*P*<0.0001) and by 97.8% with the DEA-C3D/colistin combination treatment (*P*<0.0001). There was no statistically significant difference in mean biofilm biomass or mean surface area coverage between colistin treatment alone and treatment with the DEA-C3D/colistin combination, though there was a statistically significant change in mean maximum biofilm thickness (51.5% reduction, *P=*0.0193).

## Discussion

Chronic *P. aeruginosa* biofilm infection in the CF lung is associated with worsening respiratory status, and current antibiotic therapies are ineffective; our study has shown that the NO-releasing cephalosporin prodrug DEA-C3D is effective at dispersing such biofilms *in vitro*.

Previous studies with DEA-C3D had demonstrated its ability to disperse biofilms formed by the *P. aeruginosa* laboratory strain PAO1.^11^^,^^12^ In these studies, clinical *P. aeruginosa* strains were not examined and biofilms were only grown for 6 h. During the early stages of biofilm formation, planktonic cells attach to a substratum, first reversibly and then irreversibly. Cells then begin to produce a self-encasing extracellular matrix and, at later stages of biofilm maturity, microcolony structures.^19^ A 6 h incubation period is likely to recapitulate only the very early stages of biofilm formation. In this study, biofilms were cultured for a total of 44 h and are thus more representative of mature biofilms. Established biofilms are more difficult to eradicate than nascent biofilms and are likely to better represent chronic *P. aeruginosa* infection *in vivo*.^20^

Before performing the biofilm experiments, a highly sensitive chemiluminescence method was used to confirm release of NO from two variants of DEA-C3D, i.e. the potassium salt and free carboxylic acid forms, following exposure to a commercial β-lactamase (*B. cereus* penicillinase). We noted that the DEA-C3D potassium salt released almost double the amount of NO compared with the free acid. The reasons for the difference are not clear as both forms would be present in the buffered aqueous solution after equilibration. Differences in aqueous solubility may be a factor, where the potassium salt would be expected to be more soluble.^21^ The importance of this observation for potential clinical applications of C3Ds will require further investigation. Due to its greater propensity to generate NO under *in vitro* experimental conditions, the DEA-C3D potassium salt form was selected for use in the biofilm experiments.

Biofilms formed by clinical isolates taken from adolescent and adult CF patients with chronic *P. aeruginosa* respiratory infections were dispersed by DEA-C3D but the reductions in biofilm biomass varied between isolates. At the highest concentration tested, PAO1 biofilms showed a 52% reduction in biomass following treatment, while the clinical isolates displayed reductions between 15% and 45%. One possible explanation for this could be differing levels of β-lactamase expression by each strain, leading to variations in NO exposure. Although encoded for chromosomally, β-lactamase AmpC is expressed at low basal levels in environmental strains. Stable derepression leading to a higher expression of AmpC commonly occurs in CF isolates.^22^^,^^23^ Another explanation is that NO dispersal responses vary across strains due to differences in the state of biofilm maturity. While all four *P. aeruginosa* strains were cultured for 44 h, their rates of biofilm growth were different. Further investigations are warranted to investigate these theories.

The most commonly prescribed aminoglycoside antibiotic for CF patients is tobramycin, which targets the ribosomal subunits of Gram-negative pathogens to inhibit protein synthesis.^24^ Like most antibiotics, tobramycin is only effective against actively dividing cells and is less effective against cells within a biofilm. Using CLSM and COMSTAT analysis, treatment of *P. aeruginosa* biofilms with DEA-C3D alone verified the results obtained with crystal violet assays, indicating dispersal of the biofilm. In contrast, treatment with tobramycin alone showed no effect on biofilms, consistent with findings from the literature.^10^^,^^25^ The combination of DEA-C3D and tobramycin was an effective anti-biofilm treatment, reducing the biofilm biomass over 2-fold more than tobramycin alone. These data demonstrate that biofilm cells tolerant of tobramycin-mediated killing are responsive to the NO dispersal signal. However, there was no statistically significant difference in the anti-biofilm effect of DEA-C3D treatment and treatment with the DEA-C3D/tobramycin combination.

Colistin is an antibiotic that is often administered by inhalation to treat *P. aeruginosa* infection in CF patients.^26^ Using CLSM, we demonstrated that colistin treatment of *P. aeruginosa* biofilms had affected the viability of a large proportion of the biofilm cells and had reduced the mean biofilm biomass by ∼89.8% in comparison with the untreated control. However, multiple dense aggregates or microcolony-like structures containing dense clusters of live cells were still present. Co-treatment with colistin and DEA-C3D also showed an anti-biofilm effect, with a 97.8% reduction in mean biofilm biomass in comparison with the untreated control. However, quantitative analysis revealed that there was no significant difference in total biofilm biomass or surface area coverage between the colistin-only and DEA-C3D/colistin treatments.

Interestingly, however, the mean maximum biofilm thickness was reduced from ∼32 μm (untreated control biofilms) to ∼22 μm with DEA-C3D treatment, to ∼14 μm with colistin treatment and to <7 μm with the DEA-C3D/colistin combination. This reduction in biofilm thickness with the DEA-C3D/colistin combination treatment compared with the colistin-only treatment was statistically significant. This can be attributed to the scarcity of the large clusters of live cells in the biofilms treated with the combination treatment, which were seen in abundance in the biofilms treated only with colistin. These microcolony structures likely represent a subpopulation of cells more tolerant to colistin attack and/or may be classed as ‘persister’ cells. Persister cells often represent <1% of the biofilm population, are more difficult to eradicate than the other 99% and their presence can cause repopulation or regrowth of a biofilm which is one of the known mechanisms of biofilm tolerance.^27^ Therefore, the reduced biofilm thickness and the reduced number of large clusters of live cells with the DEA-C3D/colistin combination indicates that this is a superior treatment to colistin alone and may have an increased likelihood of preventing biofilm regrowth following cessation of treatment. Further investigations are required to support this theory.

In conclusion, we have demonstrated that DEA-C3D is an effective anti-biofilm against CF isolates of *P. aeruginosa in vitro*. Though we could not confirm any synergistic activity of DEA-C3D in combination with tobramycin or colistin, we observed evidence to suggest that DEA-C3D in combination with colistin could prevent biofilm regrowth. Current efforts in our labs are aiming to create β-lactamase-stable C3Ds designed to release NO and disperse *P. aeruginosa* biofilms following reactions with transpeptidases. Such C3Ds are potentially dual-action anti-biofilm compounds able to both kill bacteria and disperse biofilms, thus removing the requirement for co-administered antibiotics.

## Supplementary Material

dkz378_Supplementary_Data
