# Supplementary material for: Cephalosporin nitric oxide-donor prodrug DEA-C3D disperses biofilms formed by clinical cystic fibrosis isolates of Pseudomonas aeruginosa
Source: J Antimicrob Chemother. 2019 Sep 17;75(1):117–25. doi: 10.1093/jac/dkz378 (PMC6910178; doi:10.1093/jac/dkz378)
Supplement: dkz378_Supplementary_Data [file dkz378_supplementary_data.docx]

**Supplementary data**

a) b)

c) d)

**Figure S1.** Planktonic growth in supernatants from dispersal assays in Fig 3; PAO1 (a) and three CF isolates of *P. aeruginosa*, PA21 (b), PA30 (c), and PA68 (d). Planktonic values for cultures treated with DEA-C3D are either the same, or significantly higher than the untreated control, indicating lack of direct antibacterial action and the occurrence of biofilm dispersal leading to increased planktonic cells. Absorbance values were normalised based on the untreated control group (0 μM) for each strain. Results represent the mean ±SEM from two independent experiments, each with 6 technical replicates. Ordinary one-way ANOVA with Dunnett’s multiple comparisons test was used for statistical analyses and each treatment group was compared to the untreated control.


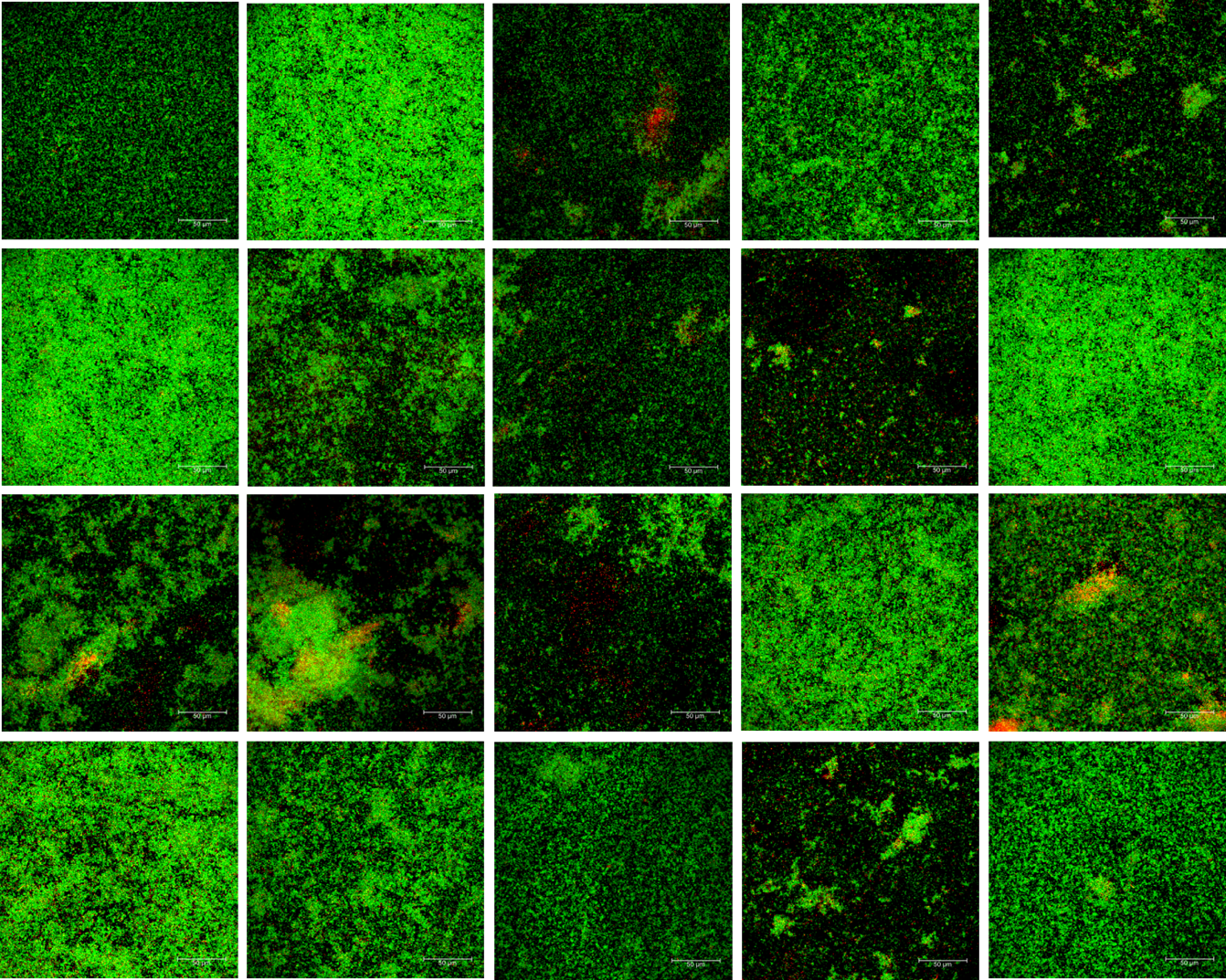


**Figure S2.** All twenty replicate CLSM images taken of untreated control biofilms (n = 4), stained with SYTO9 and PI, and in relation to data shown in Figures 4 and 5. Images shown are maximum projection images (cumulative z stacks); x and y axes = 246 μm by 246 μm (scale bar = 50 μm).


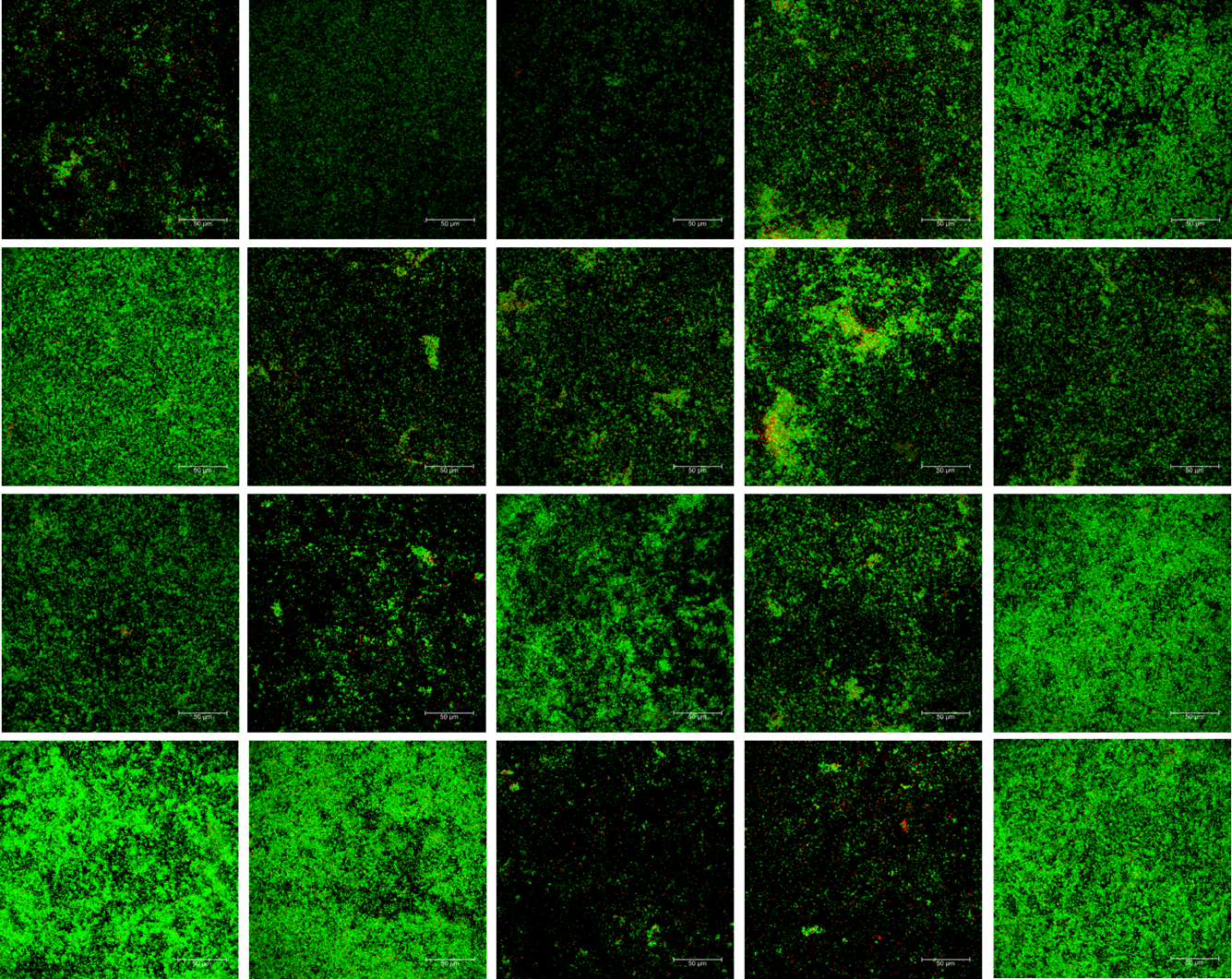


**Figure S3.** All twenty replicate CLSM images taken of biofilms treated with 256 μM DEA-C3D (n = 4), stained with SYTO9 and PI, and in relation to data shown in Figures 4 and 5. Images shown are maximum projection images (cumulative z stacks); x and y axes = 246 μm by 246 μm (scale bar = 50 μm).


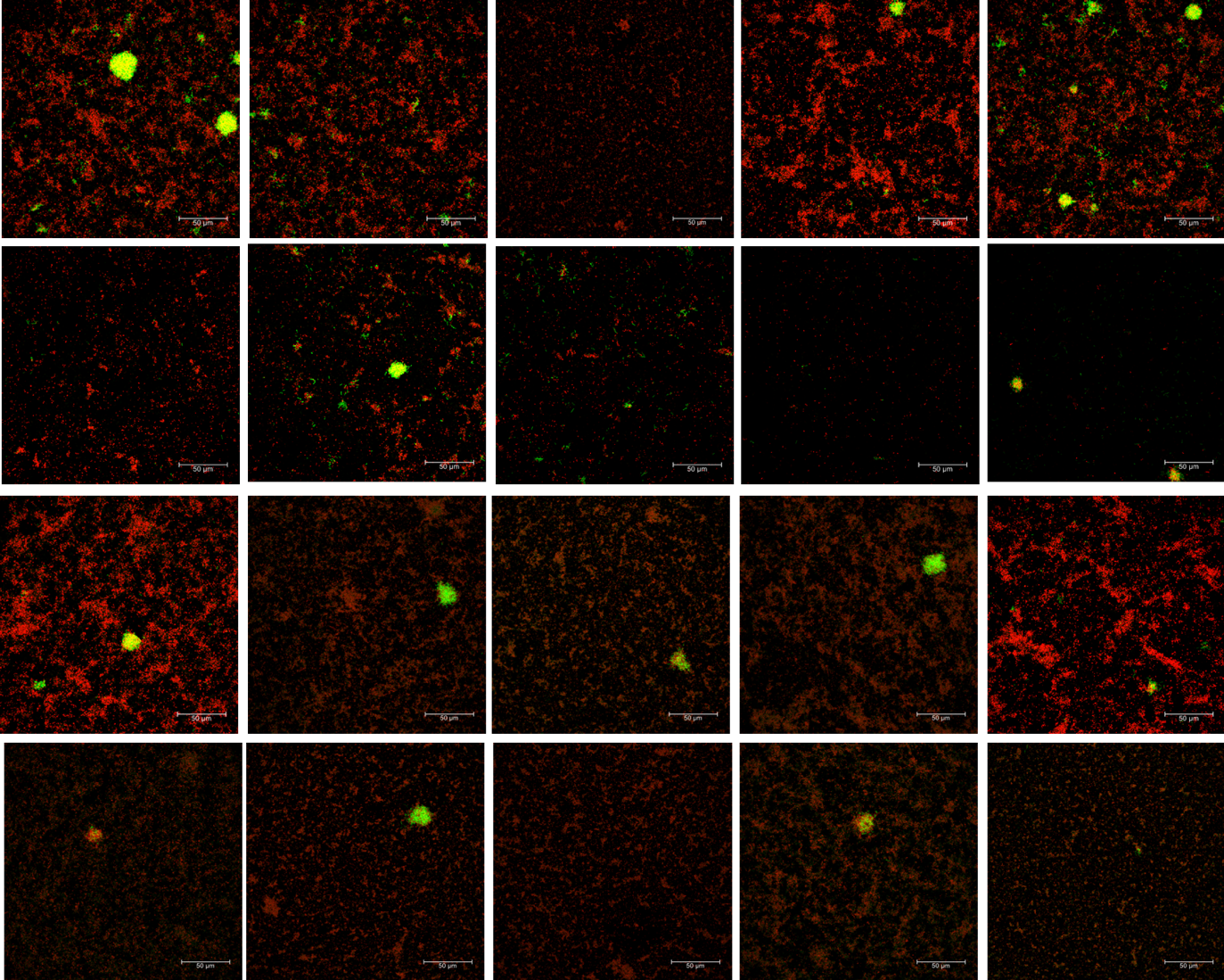


**Figure S4.** All twenty replicate CLSM images taken of biofilms treated with 16 μg/ml colistin (n = 4), stained with SYTO9 and PI, and in relation to data shown in Figures 4 and 5. Images shown are maximum projection images (cumulative z stacks); x and y axes = 246 μm by 246 μm (scale bar = 50 μm).


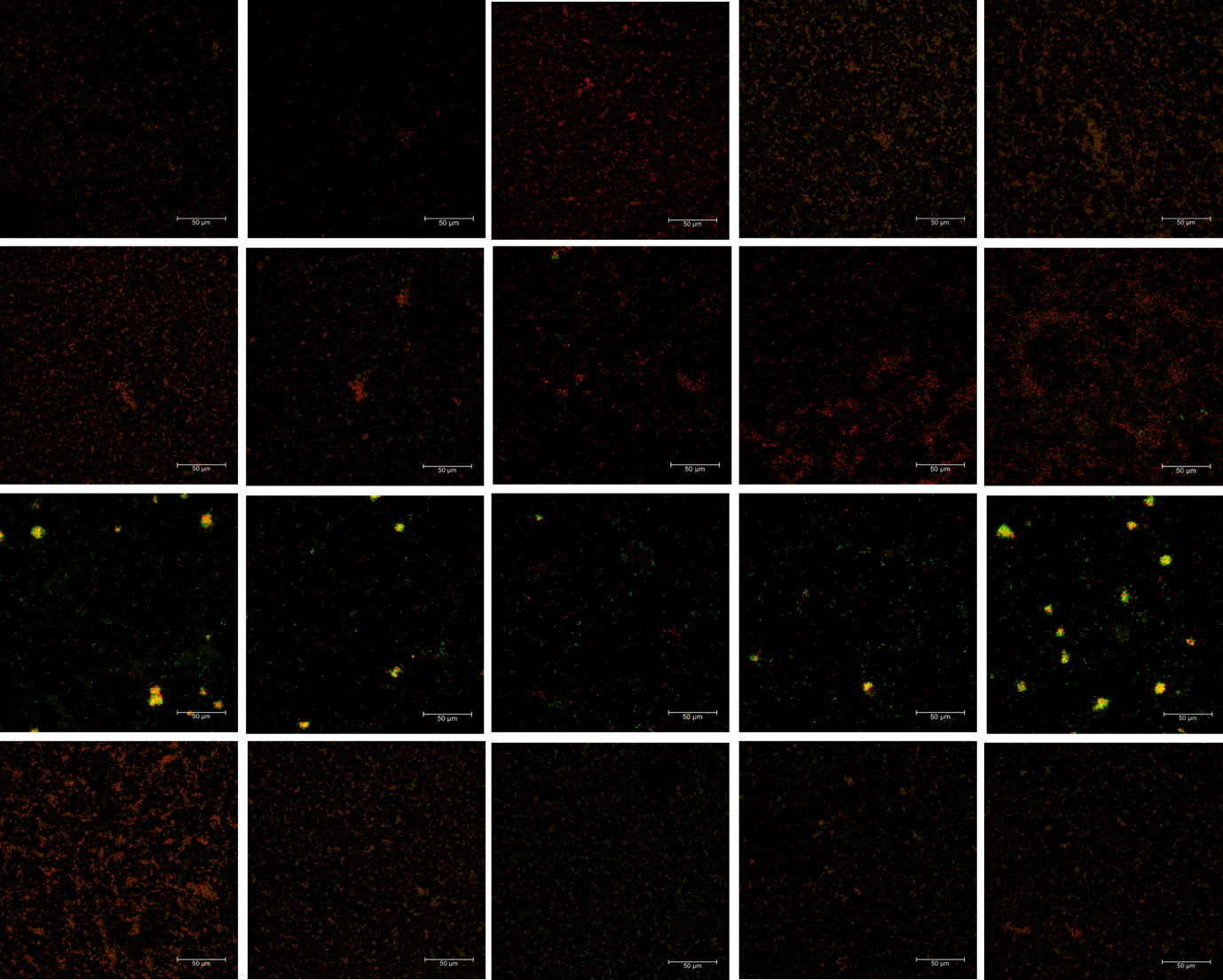


**Figure S5.** All twenty replicate CLSM images taken of biofilms treated with the DEA-C3D-colistin combination (n = 4), stained with SYTO9 and PI, and in relation to data shown in Figures 4 and 5. Images shown are maximum projection images (cumulative z stacks); x and y axes = 246 μm by 246 μm (scale bar = 50 μm).
